# E-cigarette or Vaping product use Associated Lung Injury (EVALI) in a 15 year old female patient – case report

**DOI:** 10.1186/s13052-022-01314-6

**Published:** 2022-07-19

**Authors:** Chiara Casamento Tumeo, Alessandra Schiavino, Maria Giovanna Paglietti, Francesca Petreschi, Alessandra Ottavianelli, Alessandro Onofri, Claudio Cherchi, Paolo Tomà, Renato Cutrera

**Affiliations:** 1 Academic Department of Pediatrics (DPUO), Bambino Gesù Pediatric Hospital, IRCCS, Rome, Italy; 2 Pediatric Pulmonology & Respiratory Intermediate Care Unit, Sleep and Long Term Ventilation Unit, Academic Department of Pediatrics (DPUO), Bambino Gesù Pediatric Hospital, Rome, Italy; 3 Department of Diagnostic Imaging, Oncological Radiotherapy and Hematology, Fondazione Policlinico Universitario A. Gemelli, IRCCS, Rome, Italy; 4 Department of Imaging, Bambino Gesù Pediatric Hospital, IRCCS, Rome, Italy

**Keywords:** EVALI, E-cigarette, E-cigarette or Vaping product use Associated Lung Injury, THC, Case report

## Abstract

**Background:**

E-cigarettes are devices which allow to aerosolize liquids containing nicotine or other substances. Ever since they were released on the market in 2006, the number of users have been constantly increasing, especially among adolescents, ranging from 7,6% to 9,3% in the age group 18–24 years old from 2014 to 2019. Hand in hand with the spread of E-cigarettes many have been the efforts to understand their impact on health. EVALI (E-cigarette or Vaping product use Associated Lung Injury) is an emerging condition with a heterogeneous presentation with several reported cases worldwide. We mean to report a case of EVALI in a 15-year-old female Caucasian patient, who's currently attending her clinic follow-up at Bambino Gesù Pediatric Hospital in Rome.

**Case presentation:**

The patient was admitted to the Emergency Room due to acute respiratory failure in November 2020. At admittance, she was severely dyspneic (HR 120 bpm, SatO2 75%). As she was hospitalized amid the COVID-19 pandemics, she underwent a nasopharyngeal swab for SARS-CoV2, which turned out negative, and a chest CT scan. Chest CT scan showed a central ground grass pattern with peripheral sparing. At the anamnestic recall, it was disclosed she was an e-cigarette smoker and occasional marijuana user. The microbiological work-up proved only positive for Rhinovirus. Her clinical and radiological case was discussed with our radiologist who suspected EVALI. She was assisted through HFNC, antibiotical therapy and corticosteroids with a dramatic recovery within the first 48 h.

**Conclusions:**

EVALI started being recognized a specifically nosological entity in summer 2019, with increasing cases being reported. No diagnostic criteria have been agreed upon yet, but its usual presentation includes respiratory, gastrointestinal and systemic symptoms of different degree and the diagnosis can be hypothesised in case the patient has an evocative clinical and radiological presentation and has been an E-cigarette smoker in previous 90 days. Due to the novelty of the condition and its heterogeneous presentation it is of interest to report the cases in which EVALI is identified to raise awareness about this emerging new-age disease.

## Background

Electronic cigarettes are battery-powered devices which allow to inhale vapours coming from the heating of a liquid (e-liquid) containing nicotine and other substances, flavourings, and additives. Once the device is switched on, the heating element at the bottom heats up the liquid turning it into an aerosol that's inhaled and exhaled by the user. Electronic cigarettes were approved for use in Europe in 2006 and in the United States in 2007. According to one narrative review, the prevalence of current E-cigarette use ranges from 0,2 to 27%, ever-use ranges from 5,5% to 56,6% and daily use ranges from 1% to 2,9% [1]. According to the National Health Interview Survey (NHIS) data in the United States the prevalence of e-cigarette use increased from 2014 to 2019 from 3,7% to 4,9% and, more in detail, it increased from 7,6% to 9,3% in the age group 18–24 years old [2].

According to ISTAT data, E-cigarette use in Italy increased from 2014 to 2019 from 1,5% to 2,4% [3], while according to the Global Youth Tobacco Survey led in 2018 in Italy, what emerged from the survey on 1700 students is that E-cigarette use increased from 0% in 2010, to 8,4% in 2014 to 17,5% in 2018; 21,9% of users being male and 12,8% being females [4]. To be accounted as reasons behind the constant growth in prevalence of E-cigarette use there are the believes that E-cigarettes are less harmful than traditional tobacco cigarettes, that they could help tobacco-smokers quit the smoking habit and the several flavours that represent one of the most appealing factors to adolescents.

Hand in hand with the spread of electronic cigarettes, there have been many attempts to understand the present and future health concerns behind the use of E-cigarettes. What emerged indeed, refuting the false idea that these devices could have been used as a safe alternative to tobacco, is that the exposure to the E-vapours would put the user in contact with many chemicals (such as diethylene glycol and formaldehyde) with several potential health risks. The exposure to nicotine and the above-mentioned vapours makes E-cigarettes unsuitable for pregnant women and apparently the risk for long-term cardiovascular complications doesn’t seem to be reduced. [5] Furthermore vapours and chemicals from E-cigarette appeared to be an important trigger for asthmas, due to their irritative action on airways [6]. It’s also important to underline how E-cigarettes are relatively new devices and further studies will be needed to define long-term health consequences linked to their use.

E-cigarettes can be legally bought by users over the age of 18, but many devices come from an illicit market where one or more components of the device – such as the e-liquid or the battery – are hacked for recreational use, exposing the user to a variable and unpredictable cocktail of chemicals [7]. Among the most hacked component, there’s the e-liquid being substituted with liquids containing THC.

Ever since they’d been released on the market, increasing cases of E-cigarette users presenting with a heterogeneous court of respiratory, gastrointestinal and systemic symptoms started being reported. In summer 2019 EVALI was identified and defined as a new distinct emerging condition [8]. More than 2800 cases of EVALI have been reported to the Centres for Disease Control and Prevention (CDC) and among those there have been 68 deaths [9]. Most cases involved the use of both nicotine and THC containing products, generally smoked through hacked devices [10].

The diagnosis remains a diagnosis of exclusion, but the CDC, as well as the New York State Department of Health, in conjunction with the University of Rochester Medical Center have proposed a diagnostic algorithm which includes an evocative history of E-cigarette use in the last 90 days, a compatible clinical presentation with respiratory, gastrointestinal or systemic symptoms and suggestive radiological findings [11].

Due to its heterogeneous presentation, it is of interest to report those cases in which EVALI was diagnosed in order to raise awareness about this new-age emerging disease.

## Case presentation

**Fig. 1:**
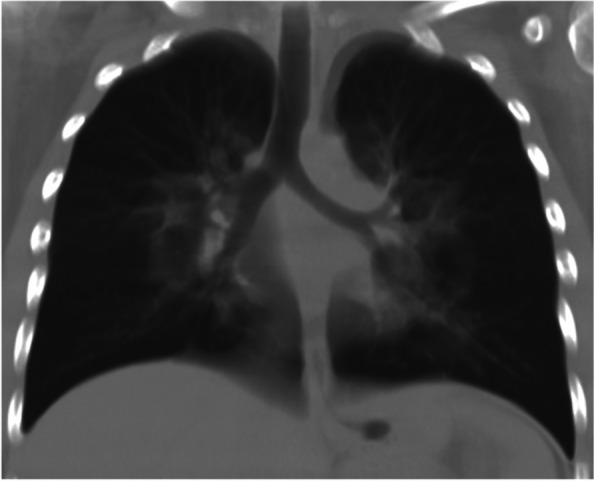
Patient’s CXR showing a central perihilar ground glass

A 15-year-old Caucasian female with a history of smoking (10 cigarettes/day), multiple allergies and asthma was admitted to the Emergency Room due to respiratory failure in November 2020, following a four-days-history of dyspnoea associated with dry cough and no fever. She’d never been on treatment for her asthmatic condition due to partial improvement in adolescence and a low familiar compliance. She was started at home on amoxicillin-clavulanic acid by her general practitioner, but spontaneously decided to stop the antibiotical therapy after three days as she saw no improvement. At the admission, she presented severely dyspnoeic with a SatO2 of 75% and she was started on supplemental oxygen support at a flow rate of 3 l/min. Bilateral wheezes with prolonged expiration were present at lung auscultation. Her bloodwork showed WBC 20.000 cells/mcl (89% neutrophils) and a CRP of 0,4 mg/ml. As she was evaluated amid the COVID-19 pandemics, she underwent a first nasopharyngeal swab for SARS-CoV2 PCR (polymerase chain reaction) assessment, which turned out negative. Her chest CT (performed at another hospital, where she was first admitted) showed a central ground glass pattern with peripheral sparing (Fig. 1).

She was started on empirical antibiotic therapy with clarithromycin and ceftriaxone, initially intravenously and subsequently orally. She was also started on HFNC, systemic steroid and aerosol with salbutamol, ipratropium, and corticosteroids. Her clinical conditions improved within the first 48 h, with progressive reduction on oxygen support. At a second evaluation performed after 24 h, bloodwork reported WBC 14,710 cells/ µl (neutrophils 85%) and a CRP of 2,6 mg/dl. In consideration of her clinical presentation, the following microbiological tests were performed: nasopharyngeal swab for respiratory viruses, M. pneumoniae, C. pneumoniae, chronic germs and a Quantiferon-TB Gold test for MBT (Mycobacterium tuberculosis) and a second nasopharyngeal swab for SARS-CoV2; the exams only came out positive for Rhinovirus, with absence of significant microbiological evidence explaining the radiological findings.

At the anamnestic recall, the patient revealed to be an e-cigarette smoker and an occasional marijuana user. Her mother reported her last joint was at the end of October and the patient disclosed she’d discontinued e-cigarette use one month prior to hospital admission.

Upon completion, further tests were performed: pulmonary function testing showed a restrictive pattern, without bronchodilator reversibility. Bronchial eNO (exhaled nitric oxide) was 3 ppb (normal reference value: < 20 ppb) and nasal eNO was slighlty reduced, with a probable influence of Rhinovirus on the latter result. The 6MWT (six-minute-walk-test) showed a slightly reduced functionality. It's significant to specificy both for the pulmonary function testing and 6MWT that the patient was slightly overweight with a sedentary lifestyle.

Furthermore, in order to re-evaluate the history of multiple allergy to inhalant allergens, total IgE and specific IgE were tested with evidence of raised total IgE 419 kU/l (normal reference value: < 100.0 kU/l). Before discharge, given the clinical stability, six days after the admission, a chest radiograph was performed in order to monitor the radiologic improvement. The chest radiograph showed bilateral hilar opacities (virtually superimposable to the CT findings). A final bloodwork assessment, performed 7 days later, showed WBC 16,120 cell/ml, neutrophils 12,240 cells/ µl, lymphocites 2340 cells/ µl, CRP 0,06 mg/dl. She was discharged after 7 days with a planned follow-up at the pulmonary clinic after one month in order to assess the clinical-radiological evolution of the case. She was also prescribed with prophylactic aerosol bronchodilator/corticosteroid and antileukotriene. In total, she followed a 14-day course of clarithromycin and a 7-day course of third generation intravenous cephalosporin. Due to the presence of acute respiratory failure, evocative imaging (“central ground grass pattern with peripheral sparing”), the history of vaping, absence of significant microbiological evidence and signs of aspecifical inflammation the diagnosis of EVALI was made. At the clinic follow-up one month later, the girl reported she's stopped smoking tobacco and e-cigarette. She reported she'd been following her prophylactic therapy as prescribed.

A toxicological exam on urine was performed, turning out negative.

Blood exams showed WBC 7580 cells/ µl, neutrophils 4500 cells/ µl, lymphocites 2340 cells/ µl, CRP was negative, renal, hepatic function and electrolytes were normal. As a first immunological assessments, total IgA, IgM, IgG, lymphocites and IgG subsets were tested: all results were in the norm. A sweat test was also performed, turning out negative. Complimentarily to the microbiological exams that were performed during hospitalization, serology for C. Pneumoniae and M. Pneumonia was done as well, with a positivity in IgG for M. Pneumoniae. The patient's case was discussed with a rheumatologist, infectivologist and radiologist. It was decided to schedule a new appointment in order to repeat the CXR (chest X-ray) scan imaging if in good health. The therapy with bronchodilator/corticosteroid was prolonged.

### Patient perspective

The patient reported a significant improvement 48 h after she’d started the treatment.

Her mood improved along as her willing to communicate and disclose important information (such as marijuana abuse) and be active part of the healing process. She did not report any adverse reaction from the treatment.

## Discussion and conclusions

In the past few years, ever since EVALI was identified as a specifical condition, many have been the efforts to define diagnostic criteria, its pathogenesis, and predisposing factors.

Looking at the 2800 cases reported to the CDC [9], approximately 66 percent of patients were male, and nearly 80 percent were under 35 years old (range 13 to 85 years) [9]. Furthermore, 22 percent of patients had underlying asthma [12] and so did our patient. Diagnostic criteria haven't been agreed upon yet and EVALI still remains a diagnosis of exclusion. The CDC, as well as the New York State Department of Health, in conjunction with the University of Rochester Medical Center have proposed a diagnostic algorithm which includes an evocative history of E_cigarette use in the last 90 days, a compatible clinical presentation with respiratory, gastrointestinal or systemic symptoms and suggestive radiological findings [11].

**Fig. 2:**
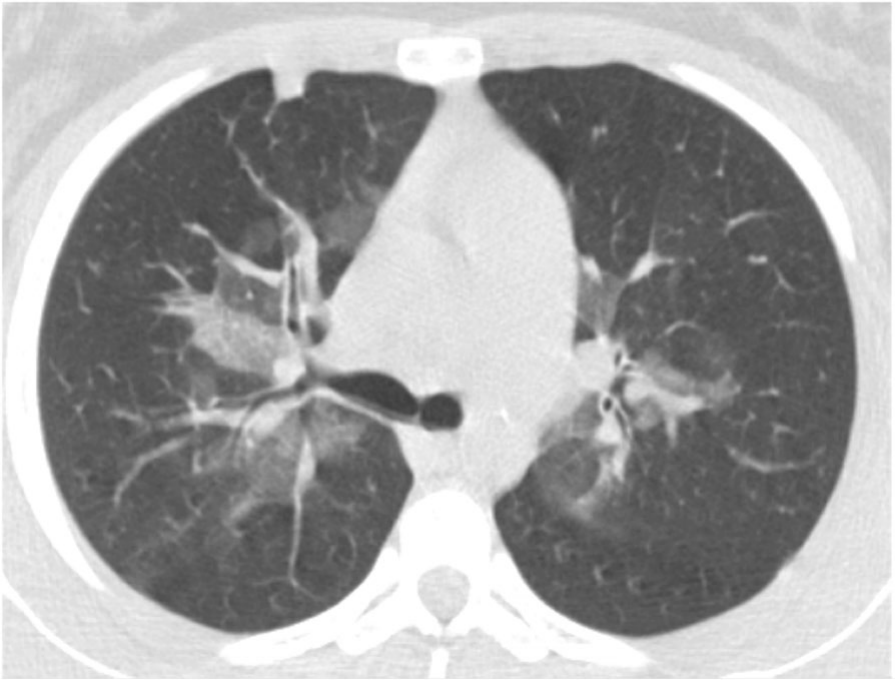
Chest CT (axial view) confirmsing the central ground glass. A small consolidation is evident anteriorly on the right

**Fig. 3:**
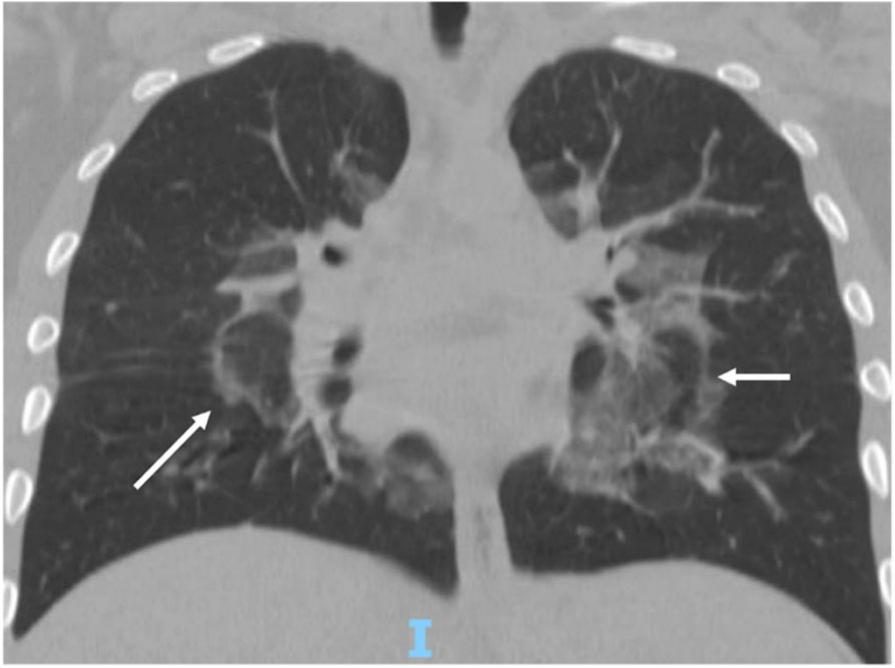
Chest CT (coronal view) showing the “atoll sign” or “reversed halo sign” bilaterally (arrrows)

Specifically, EVALI should be suspected in a patient with a history of vaping presenting with respiratory symptoms such as shortness of breath, cough, chest pain, pleuritic chest pain and haemoptysis and/or gastrointestinal symptoms such as nausea, vomiting, diarrhoea and abdominal pain. Systemic symptoms such as fever, tachycardia and tachypnoea may be present. Hypoxemia may be present and progression to respiratory failure is common [12]. Bloodwork doesn’t show specifical evidences, nevertheless, some elements may be evocative of EVALI: a non-specific raise of the inflammation indexes may be detected. Blood exams may also be useful to exclude other processes in the differential diagnosis [13, 14]. The radiological evaluation in patients with suspected EVALI generally shows diffuse hazy or consolidative opacities at the chest radiograph. Chest CT would typically show a ground glass imaging with spare of the sub pleural space [15]. GGOs (Ground-glass opacification) are common, and the most useful imaging features to help differentiate EVALI from COVID-19 are subpleural sparing of pulmonary opacities (GGOs) and centrilobular nodules (infrequent in COVID-19) (Fig. 2). In our case there was no thickening of the interlobular septa, but the reversed halo sign was present (atoll sign) (Fig. 3), which is characterized by the presence of a central ground-glass opacity surrounded by dense consolidation of crescentic shape or complete ring (sign present in late phase COVID-19 and typical of BOOP (bronchiolitis obliterans organizing pneumonia) and Wegener). These findings are consistent with diffuse alveolar damage, as seen in acute respiratory distress syndrome. The radiological findings vary according to the underlying histopathological process. Histology isn’t routinely performed, several pathologic patterns of lung injury have been reported in the setting of vaping such as lipoid pneumonia, diffuse alveolar damage, acute eosinophilic pneumonia, organizing pneumonia, diffuse alveolar haemorrhage, respiratory bronchiolitis interstitial lung disease, hypersensitivity pneumonitis and giant cell interstitial pneumonia, prompting the heterogeneity of the underlying disease processes. [16–21]. At the broncho alveolar lavage fluid (BAL) of affected patients the most commonly found substances appeared to be tetrahydrocannabinol (THC) and/or vitamin E acetate, the presence of lipid-laden macrophages seen with oil red O staining has been reported as well [22, 23]. Vitamin E had been recently looked at as possible culprit in the pathogenesis of EVALI as multiple studies report its presence in the BAL samples from patients affected from EVALI, while it hasn't been detected in healthy individuals [9, 23]. Vitamin E is often used as a thickening agent of illegal THC containing vapes. According to the American public health authorities, in order to formalise the diagnosis of EVALI, vaping should be present within 90 days prior to the symptom onset, an underlying infectious cause needs to be ruled out and there should be no evidence of other plausible diagnoses (e.g. cardiac, rheumatologic or neoplastic processes) [9, 24].

The microbiological work-up for our patient didn’t show any significant microbiological evidence explaining the radiological findings. Rhinovirus wasn’t considered to be consistent with the radiological case. There have been a few cases of acute respiratory failure due to Rhinovirus reported in literature with several radiological findings, but these were mainly in patients with comorbilities, while there are only isolated case report in immunocompetent hosts [18]. Furthermore, her familiar and personal history wasn’t consistent with any rheumatologic or specifical clinical entities to investigate. She quickly responded to therapy and more invasive exams, such as BAL, were ruled out for this case.

As we assumed that the history of multiple allergies and asthma played a role in determining the respiratory failure, we decided to investigate that possibility and the patient showed an asthmatic condition at the tests and was therefore put on preventive therapy.

Due to the personal history of CBD use and the important clinical presentation, we decided to also rule out drug use.

The first step in the treatment approach would be the cessation of vaping [24].

Antibiotic coverage is needed until an underlying infectious process is excluded.

EVALI uses improves over a course of systemic corticosteroids [25]. Oxygen support may be needed in case of worsening hypoxia or dyspnoea through an appropriate interface (nasal cannula or face masks) [26].

E-cigarettes are a relatively new device. Even though there have been many attempts to fully understand the health impact of vaping on E-cigarette users, further studies and on a longer observation time will be needed to define its actual impact on health and future long-term implications.

## Data Availability

The datasets generated and analyzed during the current study are not publicly available due to privacy but are available from the corresponding author on reasonable request, in an anonymous form.

## Abbreviations

EVALI: (E-cigarette or Vaping product use Associated Lung Injury)
NHIS: National Health Interview Survey
GYTS: Global Youth Tobacco Survey
WBC: White Blood Cell
CRP: C-reactive protein
HFNC: High Flow Nasal Cannula
THC: Tetrahydrocannabinol
CBD: Cannabinoid
CDC: Centers for Disease Control and Prevention
BT Body: Temperature
PCR: Polymerase Chain Reaction
MBT: Mycobacterium tuberculosis
eNO: Exhaled Nitric Oxide
6MWT: 6-Minute-walking-test
CXR: Chest X-ray
GGOs: Ground-glass opacification
BOOP: Bronchiolitis Obliterans Organizing Pneumonia
BAL: Broncho Alveolar Lavage
